# LncRNA TRG-AS1 stimulates hepatocellular carcinoma progression by sponging miR-4500 to modulate BACH1

**DOI:** 10.1186/s12935-020-01440-3

**Published:** 2020-08-04

**Authors:** Xuehu Sun, Yeben Qian, Xingyu Wang, Rongge Cao, Jianlin Zhang, Weidong Chen, Maoyong Fang

**Affiliations:** 1 Department of Emergency Surgery, the First Affiliated Hospital of Anhui Medical University, Hefei, 230000 Anhui China; 2 Department of Hepatobiliary Surgery, the First Affiliated Hospital of Anhui Medical University, No.218 Jixi Road, Hefei, 230000 Anhui China; 3 Department of Emergency, the First Affiliated Hospital of University of Science and Technology of China, Hefei, 230001 Anhui China

**Keywords:** TRG-AS1, miR-4500, BACH1, HCC

## Abstract

**Background:**

T cell receptor gamma locus antisense RNA 1 (TRG-AS1) has been reported to involve in the progression of glioblastoma, however the role and its underlying molecular mechanism in hepatocellular carcinoma (HCC) remain unknown.

**Methods:**

Quantitative real-time polymerase chain reaction (RT-qPCR) was applied to detect TRG-AS1 expression in HCC cells. Besides, the proliferation abilities of HCC cells were assessed by colony formation and EdU assays. The migratory and invasive abilities of HCC cells were examined by transwell assays. Imunofluorescence staining (IF) was used to analyze the epithelial–mesenchymal transitions (EMT). The interaction among TRG-AS1, miR-4500 and BTB domain and CNC homolog 1 (BACH1) were proofed by means of RIP and RNA pull down and luciferase reporter assays.

**Results:**

TRG-AS1 was conspicuously overexpressed in HCC cells. TRG-AS1 silencing apparently suppressed HCC cell proliferation, migration, invasion and epithelial-mesenchymal transition (EMT). Mechanism exploration revealed that TRG-AS1 acted as a molecular sponge of miR-4500 to regulate BACH1. MiR-4500 silencing or BACH1 overexpression in BACH1-downregulated cells fully rescued cell proliferation migration, invasion and EMT progress.

**Conclusion:**

TRG-AS1 regulates HCC progression by targeting miR-4500/BACH1 axis.

## Background

Hepatocellular carcinoma (HCC) is a type of primary liver cancer, which is regarded as the fifth common cancer worldwide [1]. There are many risk factors to implicate in this course such as chronic infection and cirrhosis by hepatitis B virus (HBV) and cirrhosis. [2]. With the development of medical technology and improvement of health system, more and more advanced therapeutic managements have been applied to treat HCC patients, such as liver transplantation [3], proton beam therapy [4], percutaneous/laparoscopy-assisted radiofrequency ablation [5] and etc. Nevertheless, the prognosis of HCC patients remains unfavorable. Therefore, exploration of mechanism associated with the initiation and progression of HCC is essential for providing a new insight for the treatment of HCC.

Noncoding RNAs (ncRNAs) are consisted of short and long ncRNAs, which are related with cancers [6]. Recent years, the underlying molecular mechanism of lncRNAs have been discussed in multiple cancers, including HCC [7, 8]. For instance, PVT1 silencing significantly suppresses prostate cancer cell growth [9]. HANR promotes tumorigenesis of HCC and enhances chemoresistance [10]. In addition, RGMB-AS1 and CDKN2B-AS1 accelerate tumor metastasis of HCC [11, 12].

LncRNAs can exert tumor-suppressing or tumor-promoting functions by sponging miRNAs to up-regulate downstream mRNAs [13]. For example, MCM3AP-AS1 plays an oncogenic role in HCC by absorbing miR-194-5p to promote FOXA1 expression in HCC [14]. T cell receptor gamma locus antisense RNA 1 (TRG-AS1) has been determined to be oncogenic in glioblastoma [15]. However, whether TRG-AS1 had the effect on HCC cellular processes remains to be unveiled.

In this study, we focused on the role and underlying mechanism of TRG-AS1 in HCC.

## Materials and methods

### Cell lines

Human liver immortalized cell line THLE-2 and human HCC cell line SK-HEP-1 were both procured from ATCC (Manassas, VA, USA). Human HCC cell line HCCLM3 was procured from China Center for Type Culture Collection (CCTCC; Wuhan, China), MHCC97-L cell line was procured from Procell Life Science & Technology Co., Ltd. (Wuhan, China). MHCC97-H and Huh-7 cell lines were purchased from the Cell Bank of the Chinese Academy of Sciences (Shanghai, China). THLE-2 cell was cultured in BEGM (Lonza/Clonetics Corporation, Walkersville, MD, USA). HCCLM3, Huh-7 and SK-HEP-1 was cultured in DMEM (Gibco, Grand Island, NY, USA) with 10% fetal bovine serum (FBS; Gibco), plus 1% penicillin/streptomycin (Thermo Fisher Scientific, Grand Island, NY, USA). MHCC97-H and MHCC97-L cells were cultured in RPMI-1640 with 10% FBS. All cells were maintained in a 5% CO_2_ incubator at 37 °C.

### Total RNA extraction and RT-qPCR

Total RNA was extracted with application of TRIzol Reagent (Invitrogen, Carlsbad CA, USA), and then was processed with PrimeScript Reverse Transcriptase Kit (Takara, Shiga, Japan). The acquired cDNA template was used for quantitative analysis with SYBR Green PCR Kit (Takara) on ABI Prism 7900HT sequence detector (Applied Biosystems, Foster City, CA, USA). At length, results were calculated by 2^−ΔΔCt^ method by using GAPDH or U6 as internal control.

### Cell transfection

The shRNAs specifically targeting TRG-AS1 and BACH1 were designed and constructed by GenePharma (Shanghai, China), together with the negative control (NC)-shRNAs. The full-length of BACH1 cDNA sequence was cloned into pcDNA3.1 vector (Invitrogen) for overexpression. The empty pcDNA3.1 vector was employed in NC group. In addition, miR-4500 inhibitor and miR-4500 mimics were synthesized by Ribobio (Guangzhou, China), along with the NCs. All these were transfected into HCCLM3 and Huh-7 cells for 48 h with application of Lipofectamine 3000 (Invitrogen). This assay contained three independent repeats.

### Colony formation assay

After transfection, HCCLM3 and Huh-7 cells were planted into 6-well plates at a density of 800 cells per well and incubated for 14 days. Then, cells were fixed by methanol for staining in 0.5% crystal violet solution (Sigma-Aldrich). The number of colonies was counted manually. This assay contained three independent repeats.

### EdU assay

transfected HCCLM3 and Huh-7 cells were collected and planted into 96-well plates (1 × 10^4^ cells per well), followed by addition of EdU assay kit (Ribobio) for 2 h at 37 °C. After that, cells were treated in DAPI solution for 5 min at room temperature. At length, fluorescence microscope (Olympus, Tokyo, Japan) was used for detecting proliferative cells. This assay contained three independent repeats.

### Transwell assay

2 × 10^4^ cells in serum-free medium were prepared and added into the upper chamber of Transwell inserts (24-well; Corning Incorporated, Corning, NY, USA) for cell migration assay. As for cell invasion assay, the chamber was coated with Matrigel membrane (BD Biosciences, Franklin Lakes, NJ, USA). Lower chamber was filled with 100% complete medium. Migrated and invaded cells were visualized by crystal violet staining after fixing by 4% PFA. 5 random fields were chosen and observed by optical microscope (Olympus). This assay contained three independent repeats.

### Immunofluorescence staining (IF)

Cells on the culture slides were collected after adhered to the slides, and then washed in PBS three times. After that, cells were fixed for 10 min and blocked in 5% BSA for 10 min, followed by culture with primary antibodies against E-cadherin and N-cadherin (Cell Signaling Technology, St Louis, Missouri, USA) at 4 °C overnight. Afterwards, the secondary antibody was added before DAPI staining. The stained cells were visualized using fluorescence microscope (Olympus). This assay contained three independent repeats.

### Fluorescent in situ hybridization (FISH)

The FISH probe specifically targeting TRG-AS1 was procured from Ribobio and used as guided. Cells were cultivated with the probe in the hybridization buffer, and then stained in Hoechst solution, analyzed by fluorescence microscope (Olympus). This assay contained three independent repeats.

### Nucleus-cytoplasm fractionation

The subcellular location of TRG-AS1 was also analyzed via nucleus-cytoplasm fractionation assay with application of Cytoplasmic & Nuclear RNA Purification Kit (Norgen, Belmont, CA, USA). Cell cytoplasm and cell nucleus were separated by cell fractionation buffer. After centrifugation, the RT-qPCR was conducted for the content of TRG-AS1, U6 and GAPDH were used as internal controls. This assay contained three independent repeats.

### RNA immunoprecipitation (RIP)

RIP assay was studied by use of control IgG and human Ago2 antibodies in RIP buffer. The cell lysates were prepared using RIP lysis buffer, and then cultivated with the antibodies-bound magnetic beads for 6 h. After that, the immunoprecipitates were collected for RT-qPCR analysis. This assay contained three independent repeats.

### RNA pull down

RNA pull down analysis was carried out with application of the Pierce Magnetic RNA–Protein Pull-Down Kit (Thermo Fisher Scientific, Waltham, MA, USA). Cell proteins were mixed with magnetic beads and the biotin-labeled probes for TRG-AS1 or miR-4500. After collecting the RNA–protein mixture, RT-qPCR was used for analysis. This assay contained three independent repeats.

### Luciferase reporter assay

The fragments of TRG-AS1 or BACH1 3′UTR, which contained the wild-type and mutated binding sites of miR-4500, were first prepared for inserting into the pmirGLO dual-luciferase vectors (Promega, Madison, WI, USA). After that, the acquired constructs were co-transfected into HCCLM3 and Huh-7 cells with miR-4500 mimics or NC mimics. Forty-eight hours later, the relative luciferase activity was examined by Dual-luciferase reporter assay system (Promega). This assay contained three independent repeats.

### Western blot

Total protein was extracted from HCC cells using RIPA lysis buffer. Extracted proteins were subsequently loaded on SDS–polyacrylamide gels and then transferred to PVDF membranes which were blocked in 5% non-fat milk for 1 h and then incubated with primary antibodies against E-cadherin (1:1000, ab238099, Abcam, USA), N-cadherin (1:5000, Abcam) and GAPDH (1:2000, Abcam) at 4 °C overnight. Then, the membranes were incubated with anti-rabbit secondary antibodies (1:5000; Abcam). Finally, the signals were detected on the ECL chemiluminescence system.

### Statistical analyses

Each assay of this study contained three independent repeats, and results were shown as the mean ± SD. The group difference was analyzed by one-way ANOVA or Student’s *t* test, with application of GraphPad PRISM 6 (GraphPad, San Diego, CA, USA). The experimental data were collected when p < 0.05.

## Results

### TRG-AS1 plays a tumor-promoting role in HCC

**Fig. 1:**
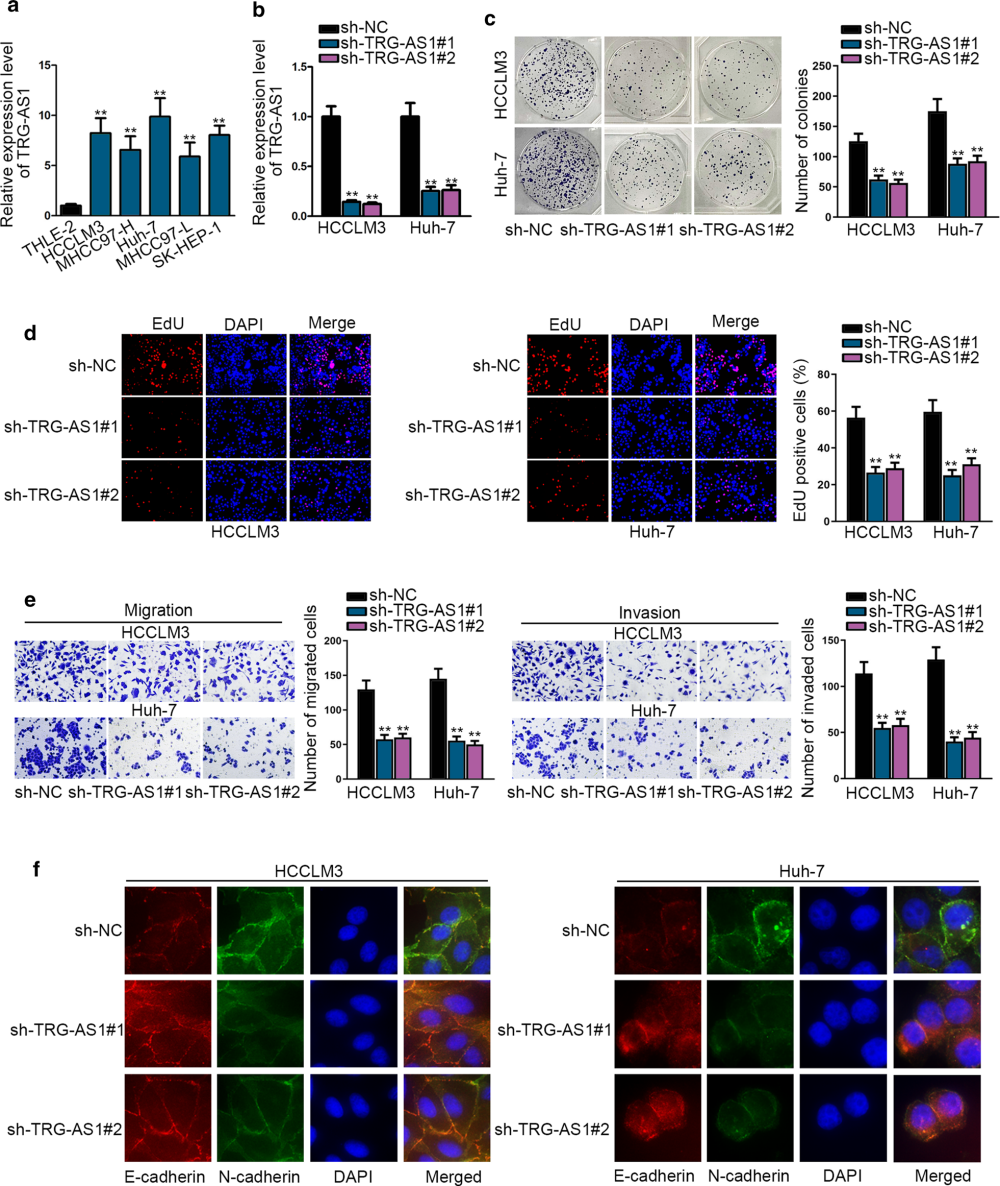
TRG-AS1 plays a tumor-promoting role in HCC. **a** RT-qPCR was used to test the expression of TRG-AS1 in HCC cell lines (HCCLM3, MHCC97-H, Huh-7, MHCC97-L and SK-HEP-1) and normal human liver immortalized cell THLE-2. **b** Transfection efficiency of sh-TRG-AS1#1/#2 in HCCLM3 and Huh-7 cells was assessed by RT-qPCR. **c** Colony formation assay was used to test proliferation ability of tested cells transfected with sh-TRG-AS1#1/#2. **d** Effects of sh-TRG-AS1 on proliferation of HCCLM3 and Huh-7 cells were detected by EdU assay. **e** Invasion and migration capabilities of both cells were examined by transwell assays, respectively. **f** Effects of TRG-AS1 depletion on EMT progress of HCCLM3 and Huh-7 cells detected by imunofluorescence staining analysis. *P < 0.05, **P < 0.01

To thoroughly investigate the role of lncRNA TRG-AS1 in HCC, we initially conducted RT-qPCR to evaluate its expression in HCC cells (HCCLM3, MHCC97-H, Huh-7, MHCC97-L and SK-HEP-1) and normal cell THLE-2. Compared with the THLE-2 cell, TRG-AS1 was obviously up-regulated in HCC cells, especially in HCCLM3 and Huh-7 cells (Fig. 1a). Then, we wondered TRG-AS1 whether affected the biological behaviors of HCC cells. RT-qPCR detected that TRG-AS1 expression was effectively cut down in the HCCLM3 and Huh-7 cells transfected with sh-TRG-AS1#1 and sh-TRG-AS1#2 compared to sh-NC group (Fig. 1b). Subsequently, we observed that TRG-AS1 knockdown effectively restrained the number of colonies and EdU-positive cells (Fig. 1c, d), which suggested that TRG-AS1 knockdown prevented the abilities of HCC cells to proliferate. Similar effects were also observed in cell invasion and migration (Fig. 1e). Furthermore, both mRNA and protein levels of E-cadherin and N-cadherin were detected in TRG-AS1-silenced HCC cells. Both levels of E-cadherin were enhanced after silencing of TRG-AS1, whereas that of N-cadherin were impeded by TRG-AS1 knockdown (Additional file 1: Figure S1A, B). The same tendency was observed through imunofluorescence staining analysis (Fig. 1f). Collectively, TRG-AS1 exerts a tumor-promoting role in HCC.

### TRG-AS1 binds to miR-4500 in HCC cells

**Fig. 2:**
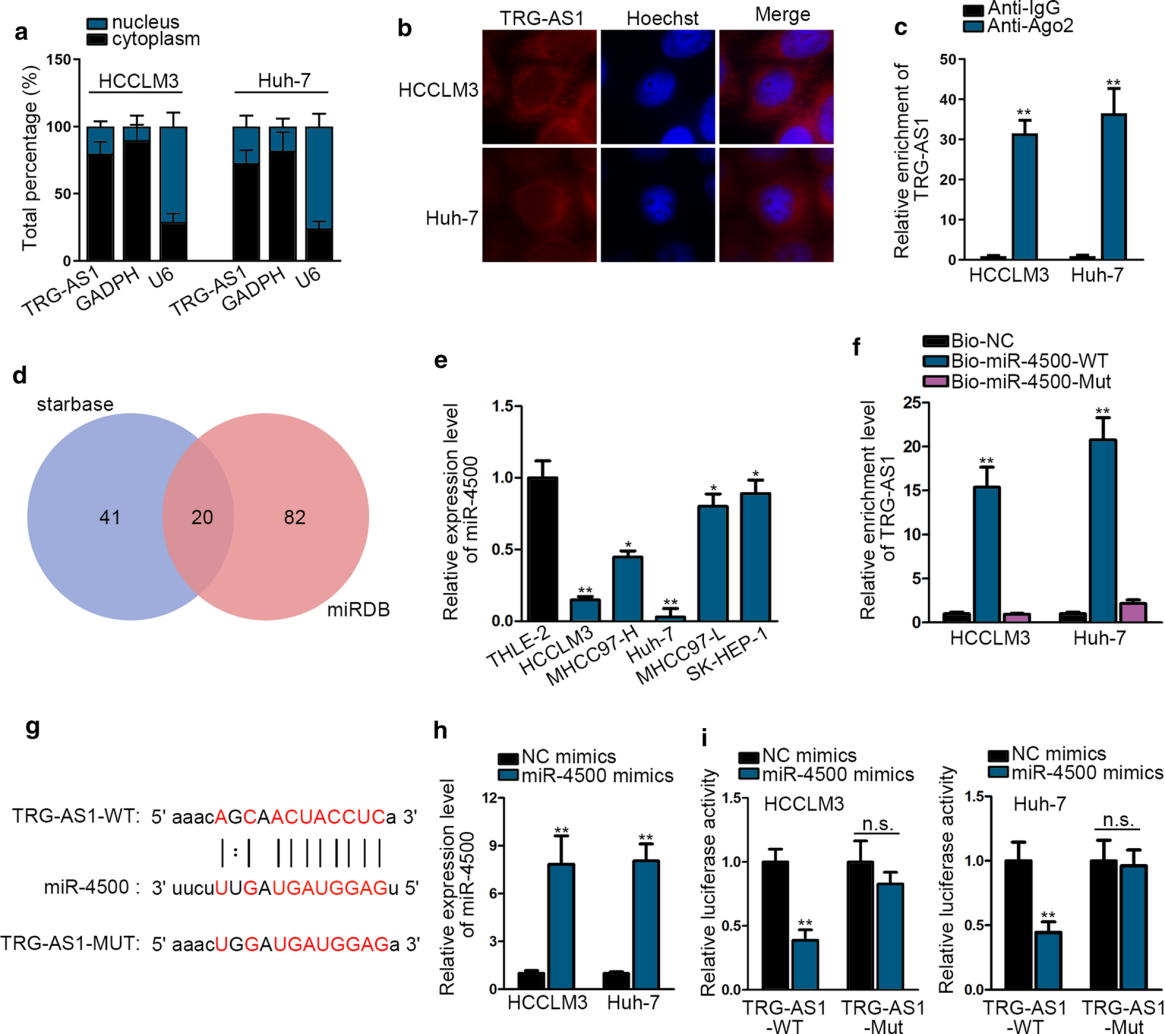
TRG-AS1 adsorbs miR-4500 in the HCC cells. **a**, **b** Subcellular fractionation assay and FISH assay evaluated the location of TRG-AS1 in HCC cells. **c** RIP assay was performed using Ago2 antibody and RT-qPCR detected the enrichment of TRG-AS1 in both HCC cells. **d** The predicted targets for TRG-AS1 binding target via miRDB and starBase. **e** RT-qPCR examined miR-4500 expression in HCC cells and THLE-2 cells. **f** The enrichment of TRG-AS1 in both HCC cells was tested by RNA pull down assay under the condition of Bio-miR-4500-WT/Mut. **g** The binding sites of TRG-AS1 to miR-4500 were predicted via ENCORI. **h** The transfection effect of miR-4500 mimics was detected via RT-qPCR. **i** Effects of miR-4500 mimics on luciferase activity of reporter gene with wild-type or mutant TRG-AS1 were observed by luciferase reporter assay. **P < 0.01

The potential mechanism associated with TRG-AS1 in HCC was investigated. Subcellular fractionation assay primarily uncovered that lncRNA TRG-AS1 was mostly distributed in the cytoplasm of HCC cells and the same result was obtained in FISH assay (Fig. 2a, b). In addition, TRG-AS1 was visibly abundant in Anti-Ago2 complex compared to Anti-IgG complex, which was confirmed by RIP assay (Fig. 2c). Based on these results, we subsequently explored the downstream targets of TRG-AS1. Firstly, we applied online databases ENCORI (http://starbase.sysu.edu.cn/) and miRDB (http://mirdb.org/) to screen out 20 miRNAs that could interact with TRG-AS1 (Fig. 2d). RNA pull down assay subsequently revealed that miR-4500 was apparently enriched in TRG-AS1 biotin probe group compared with NC biotin probe (Additional file 2: Figure S2A). As presented in Fig. 2e, miR-4500 expression was down-regulated in HCC cells. RNA pull down assay was used to further determine the relevance between TRG-AS1 and miR-4500. The results showed that the enrichment of TRG-AS1 was increased by Bio-miR-4500-WT instead of Bio-miR-4500-Mut by comparing with Bio-NC group (Fig. 2f). In addition, ENCORI also exhibited the binding sites between miR-4500 and TRG-AS1 (Fig. 2g). What’s more, miR-4500 expression was enhanced in the HCCLM3 and Huh-7 cells transfected with miR-4500 mimics (Fig. 2h). Finally, luciferase reporter assay clearly indicated that the decreased luciferase activity of TRG-AS1 wild type was induced by miR-4500 mimics by contrast with NC-mimics group (Fig. 2i). Taken together, all data explained that TRG-AS1 adsorbs miR-4500 in HCC cells.

### MiR-4500 suppresses HCC cells proliferation, invasion, migration and EMT

**Fig. 3:**
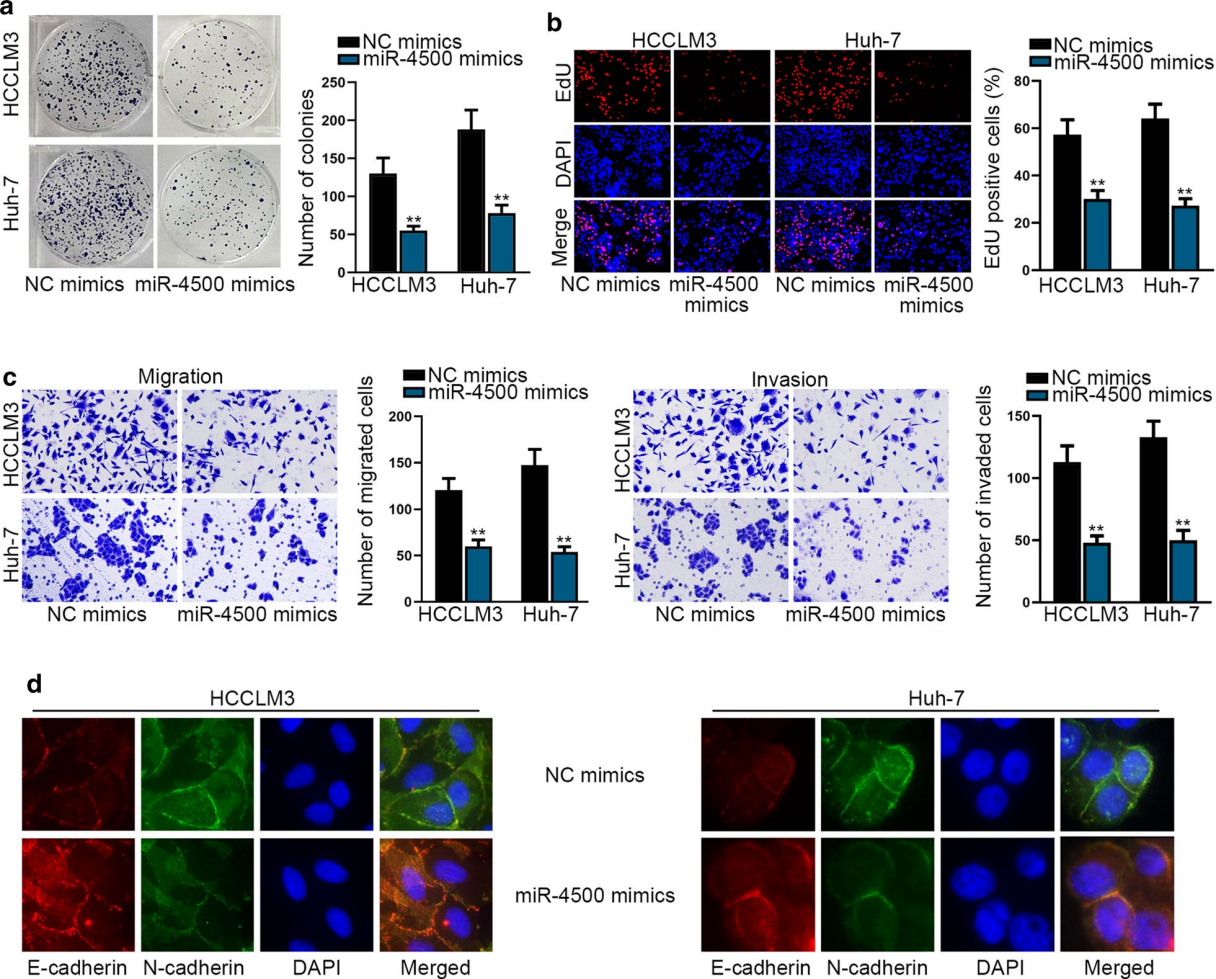
MiR-4500 suppresses HCC cell proliferation, invasion, migration and EMT. **a**, **b** Colony formation assay and EdU assay were executed to evaluate colony and proliferation capacities of HCCLM3 and Huh-7 cells in the context of miR-4500 mimics. **c** The transwell assay evaluated the invasion and migration abilities of HCCLM3 and Huh-7 cells after miR-4500 was up-regulated. **d** Under the circumstance of miR-4500 overexpression, EMT progress in HCC was measured with the assistance of imunofluorescence staining analysis. **P < 0.01

According to above data, miR-4500 was distinctly down-regulated in HCC cells. Then, we tried to explore the function of miR-4500 in HCC. As disclosed in colony formation assay and EdU assay, the number of colonies and EdU-positive cells exhibited the decreased tendency (Fig. 3a, b). Besides, the number of migrated and invaded cells were suppressed by miR-4500 mimics (Fig. 3c). Furthermore, the increased level of E-cadherin and the reduced level of N-cadherin were observed in cells with ectopic expression of miR-4500 (Additional file 3: Figure S3A, B). Meanwhile, the results of imunofluorescence staining analysis unveiled that miR-4500 overexpression reversed EMT progress by increasing E-cadherin expression and declining N-cadherin expression (Fig. 3d). Collectively, miR-4500 suppresses HCC cell proliferation and invasion and migration as well as EMT progress.

### BACH1 is modulated by the TRG-AS1 and miR-4500

**Fig. 4:**
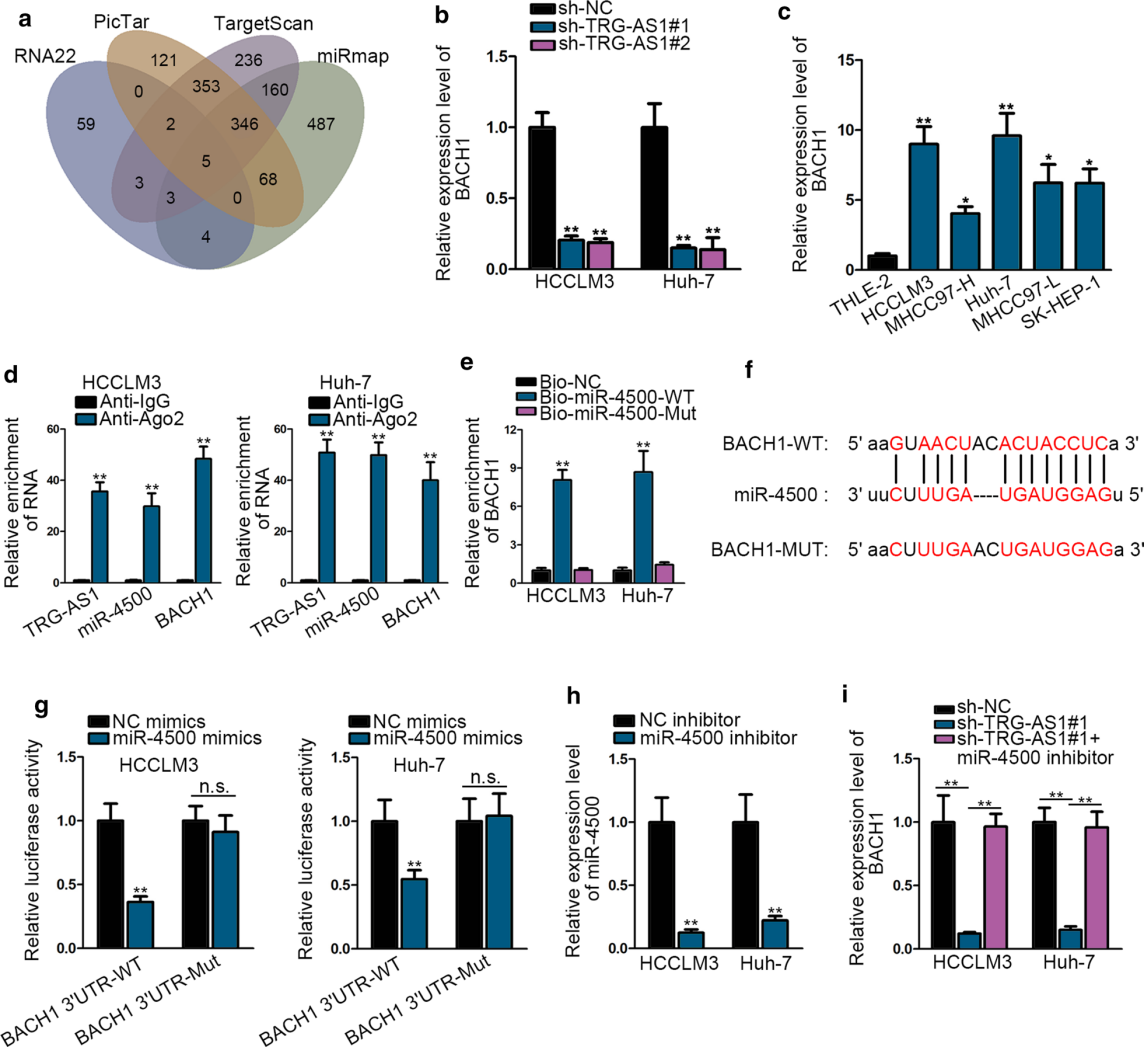
BACH1 is modulated by the TRG-AS1 and miR-4500. **a** Underlying targets of miR-4500 were obtained by RNA22, PicTar, TargetScan and miRmap databases. **b** BACH1 expression was assessed using RT-qPCR in HCC cells transfected with sh-TRG-AS1. **c** BACH1 expression in HCC cell lines and THLE-2 was examined with RT-qPCR. **d** RIP assay was performed using Ago2 antibody and the enrichment of TRG-AS1, miR-4500 and BACH1 in both HCC cells was analyzed. **e** RNA pull down assay and RT-qPCR detected the enrichment of BACH1 with the function of Bio-miR-4500-WT/Mut. **f** The binding sites were predicted between BACH1 and miR-4500 via ENCORI database. **g** Luciferase reporter assay was used to verify combination of BACH1 and miR-4500. **h** Effects of miR-4500 inhibitor on miR-4500 expression in HCC cells were detected by RT-qPCR. **i** RT-qPCR analysis was used to determine the regulatory relationship among TRG-AS1, miR-4500 and BACH1. *P < 0.05, **P < 0.01

Subsequently, we probed the possible targets of miR-4500 in HCC. There were five mRNAs selected out via RNA22, PicTar, TargetScan and miRmap datasets (Fig. 4a). And then RT-qPCR analysis manifested that BACH1 expression was observably decreased in HCCLM3 and Huh-7 cells treated with miR-4500 mimics, while others had no obvious changes (Additional file 4: Figure S4A). Moreover, BACH1 expression was also down-regulated due to TRG-AS1 silencing (Fig. 4b). We therefore detected the expression of BACH1 in HCC cells and FHC cell using RT-qPCR depending on these findings. As shown in Fig. 4c, BACH1 expression was noticeably up-regulated in HCC cells. RIP analysis showed that TRG-AS1/miR-4500/BACH1 were enriched in Anti-Ago2 complex rather than Anti-IgG complex, indicating that they were co-existed in RNA-induced silencing complex (RISC) (Fig. 4d). The results of RNA pull down suggested that BACH1 was remarkably elevated by Bio-miR-4500-WT, not by Bio- miR-4500-Mut in HCC cells (Fig. 4h). ENCORI predicted the complementary sites of BACH1 for miR-4500 (Fig. 4f). Furthermore, luciferase reporter assay uncovered that upregulation of miR-4500 dramatically cut down the luciferase activity of BACH1 3′UTR-WT, not affected that of BACH1 3′UTR-Mut compared with NC mimics in HCC cells (Fig. 4g). Additionally, RT-qPCR confirmed that miR-4500 expression was sharply silenced in HCCLM3 and Huh-7 cells treated with miR-4500 inhibitor (Fig. 4h). As exhibited in Fig. 4i, BACH1 expression was remarkably suppressed with the knockdown of TRG-AS1, and was fully recovered due to miR-4500 silence. Taken together, BACH1 is modulated by the TRG-AS1 and miR-4500.

### BACH1 acts as a tumor-promoting gene in HCC

**Fig. 5:**
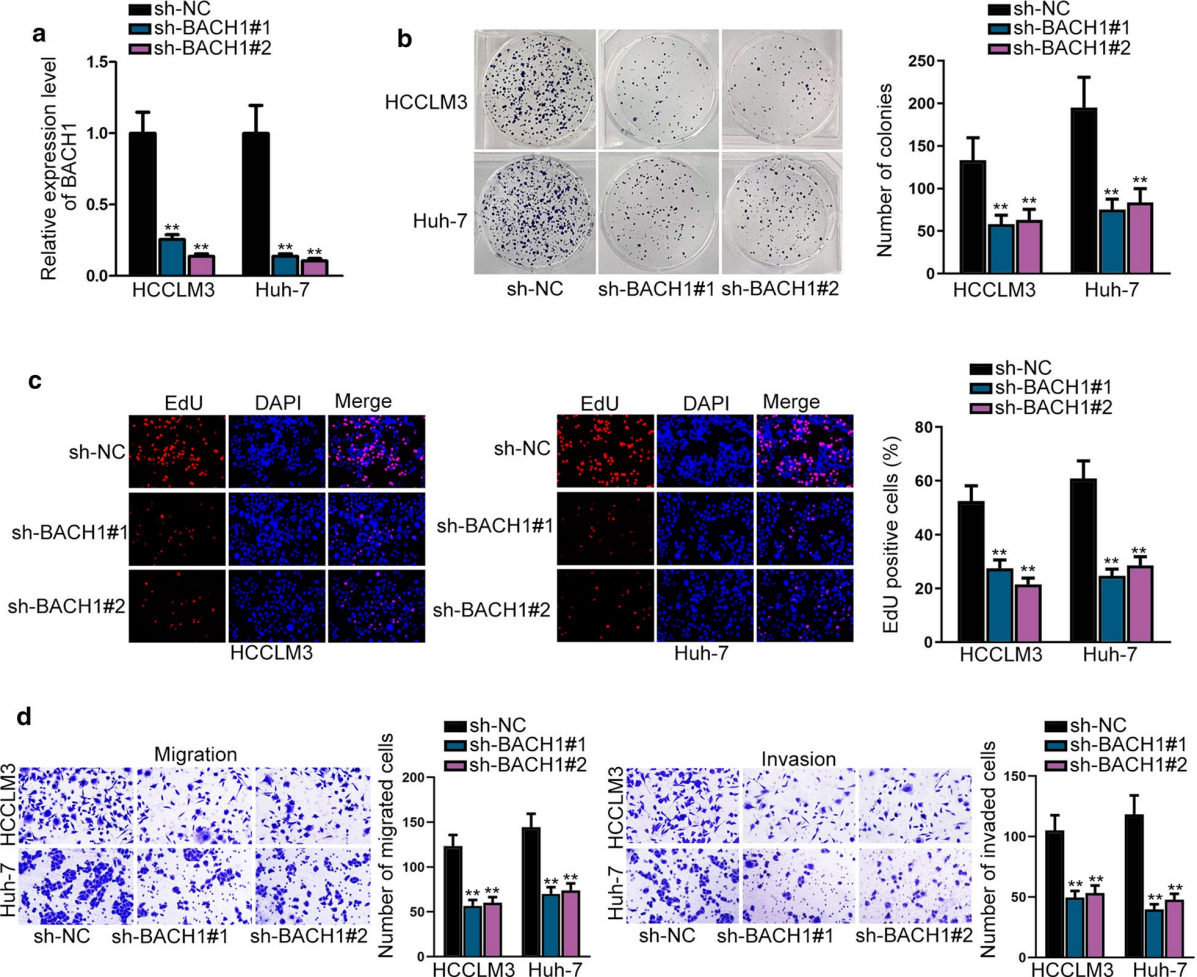
BACH1 acts as a tumor-promoting gene in HCC. **a** RT-qPCR detected BACH1 expression in transfected HCCLM3 and Huh-7 cells with sh-BACH1#1/#2. **b** The cells colony ability was assessed via colony formation assay under the situation of BACH1 knockdown. **c** HCC cell proliferation was detected by EdU assay after BACH1 was decreased. **d** When BACH1 was decreased, cell invasion and migration abilities were analyzed by transwell assays. **P < 0.01

We further explored the role of BACH1 in HCC via performing loss-of function assays. BACH1 expression was markedly silenced in HCCLM3 and Huh-7 cells treated with sh-BACH1#1/#2 (Fig. 5a). Next, BACH1 silencing resulted in the reduction of HCC cell proliferation (Fig. 5b, c). Likewise, the invasion and migration capacities of HCC cells were hampered by BACH1 down-regulation (Fig. 5d). Collectively, BACH1 contributes to HCC progression.

### TRG-AS1 contributes the progression of HCC by targeting the miR-4500/BACH1 axis

**Fig. 6:**
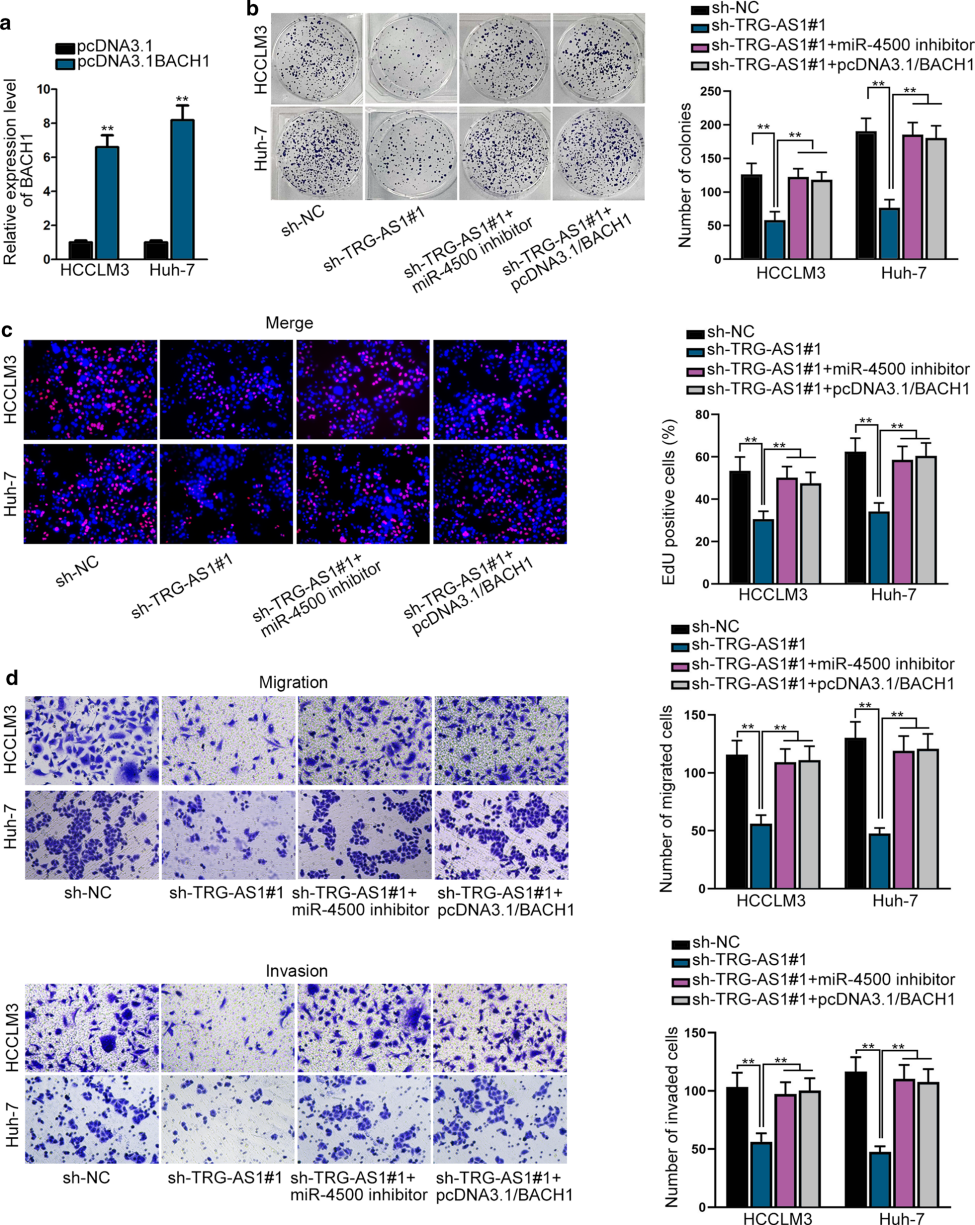
TRG-AS1 promotes HCC progression via miR-4500/BACH1 axis. **a** RT-qPCR measured the expression of BACH1 in transfected cells with pcDNA3.1/BACH1. **b** Cell proliferation ability was assessed via colony formation assay in the context of silencing TRG-AS1, or miR-4500 and up-regulating BACH1. **c** The cell proliferation change was determined following transfection with sh-TRG-AS1, miR-4500 inhibitor and pcDNA3.1-/BACH1 in EdU assay. **d** Transwell assay assessed the abilities of HCC cells invasion and migration under the same situation. **P < 0.01

To explore the interplay among TRG-AS1, miR-4500 and BACH1, HCCLM3 and Huh-7 cells were transfected with pcDNA3.1/BACH1 for rescue assays. As indicated in Fig. 6a, BACH1 was considerably up-regulated with the transfection of pcDNA3.1/BACH1. In colony formation assay, the colony formation ability of HCC cells was effectively inhibited due to TRG-AS1 knockdown, and then was fully rescued by miR-4500 down-regulation or BACH1 overexpression (Fig. 6b). Meanwhile, suppression of cell proliferation upon sh-TRG-AS1#1 could be reversed by co-transfected with miR-4500 inhibitor or pcDNA3.1/BACH1 (Fig. 6c). The reduced invasion and migration abilities caused by TRG-AS1 down-regulation could also be wholly reversed by miR-4500 silence or BACH1 increase (Fig. 6D). Furthermore, the EMT process reversed by TRG-AS1 silencing was recovered by the inhibition of miR-4500 or the overexpression of BACH1 (Additional file 5: Figure S5A-B). Overall, TRG-AS1 promotes HCC progression via the miR-4500/BACH1 axis.

## Discussion

Hepatocellular carcinoma (HCC) is one of the most highly lethal cancers. At present, authoritative researches have revealed roles of lncRNAs as promoter or inhibitor of cancer-crucial genes in HCC, which have capacity to regulate the biological behaviors of HCC cells [16, 17]. In this study, we also tried to explore the molecular mechanism underneath HCC progression.

As for TRG-AS1, it recently has been reported to regulate glioblastoma progression [15]. Given that unknown role of TRG-AS1 in HCC, thus we mainly explored the role and function of TRG-AS1 in HCC in the present study. Here, we observed TRG-AS1 presented high expression in HCC cells. In the case of TRG-AS1 knockdown, HCC cell proliferation was effectively depressed.

Epithelial-mesenchymal transition (EMT) is key and pivotal marker for the tumor development or metastasis [18–20]. In the current study, we also detected the effect of TRG-AS1 silencing on EMT process in HCC. As a result, we found that the TRG-AS1 down-regulation effectively inhibited EMT progress. All in all, the finding demonstrated TRG-AS1 exerted tumor-promoting role in HCC, which was a possible target for HCC treatment.

LncRNAs can exert functions by binding with their downstream miRNAs to further perform its regulatory effect on target mRNAs. In this study, miR-4500 was the downstream miRNA of TRG-AS1, which could interact with TRG-AS1. Gain-of-functional assays, miR-4500 overexpression inhibited HCC cell proliferation migration, invasion and EMT progress. It has been reported that miR-4500 is abnormally expressed in other human cancers. For instance, miR-4500 has tumor-suppressive function in colorectal cancer and glioma and non-small cell lung cancer [21–23]. Furthermore, this study suggested that miR-4500 targeted BACH1. BACH1, as a member of cap ‘n’ collar (CNC) and basic region leucine zipper factor family, has been reported to participate in cancer progression [24]. For instance, BACH1 plays oncogene to accelerate CRC progression and is to be a useful prognostic factor for survival and metastasis [25]. Shajari et al. show that BACH1 is a key inducer in metastasis of prostate cancer [26]. In this study, BACH1 was overly expressed in HCC cells and its down-regulation remarkably restrained HCC progression by anti-proliferation, anti-invasion/migration and anti-EMT progress. Finally, in the rescue assays, BACH1 expression was remarkably suppressed with the knockdown of TRG-AS1, and was fully recovered due to miR-4500 silencing, indicating that TRG-AS1 acted as a competitive endogenous RNA (ceRNA) by adsorbing miR-4500 to regulate BACH1. Furthermore, TRG-AS1 was able to promote HCC progression via the miR-4500/BACH1 axis.

## Conclusion

In summary, this study reported that lncRNA TRG-AS1 down-regulation inhibited proliferation, migration and invasion as well as EMT progress. TRG-AS1 may serve as a potential therapeutic target for HCC treatment.

## Supplementary information

**Additional file 1: Figure S1.** A. RT-qPCR analysis of E-cadherin and N-cadherin expression in two HCC cells transfected with sh-NC, sh-TRG-AS1#1, sh-TRG-AS1#2. B. Western blot analysis was utilized to examine the protein levels of E-cadherin and N-cadherin in HCC cells transfected with sh-NC, sh-TRG-AS1#1, sh-TRG-AS1#2. **P < 0.01.

**Additional file 2: Figure S2.** A. RNA pull down assay was performed using TRG-AS1 biotin probe and enrichment of mRNAs in both HCC cells was analyzed by RT-qPCR. **P < 0.01.

**Additional file 3: Figure S3.** A-B. The mRNA and protein levels of E-cadherin and N-cadherin were detected by RT-qPCR and western blot analyses in cells with ectopic expression of miR-4500. **P < 0.01.

**Additional file 4: Figure S4.** A. The levels of candidate mRNAs were examined by RT-qPCR in HCCLM3 and Huh-7 cells transfected with miR-4500 mimics. **P < 0.01.

**Additional file 5: Figure S5.** A-B. The mRNA and protein levels of EMT-makers (E-cadherin and N-cadherin) were measured in indicated HCC cells by RT-qPCR and western blot analyses, respectively.

## Data Availability

Not applicable.

## Abbreviations

lncRNAs: long non-coding RNAs
mRNA: messenger RNA
ceRNAs: competing endogenous RNAs
miRNA: microRNA
HCC: hepatocellular carcinoma
TRG-AS1: T cell receptor gamma locus antisense RNA 1
BACH1: BTB domain and CNC homolog 1
EMT: epithelial–mesenchymal transitions
Wt: wild-type
Mut: mutant
RT-qPCR: Quantitative real-time polymerase chain reaction
EdU: 5-ethynyl-20-deoxyuridine
DAPI: 4′,6-diamidino-2-phenylindole
FISH: fluorescent in situ hybridization
RIP: RNA immunoprecipitation
IF: imunofluorescence staining
RISC: RNA-induced silencing complex
SD: standard deviation
