# Supplementary figures and images for: LncRNA TRG-AS1 stimulates hepatocellular carcinoma progression by sponging miR-4500 to modulate BACH1
Source: Cancer Cell Int. 2020 Aug 4;20:367. doi: 10.1186/s12935-020-01440-3 (PMC7401190; doi:10.1186/s12935-020-01440-3)

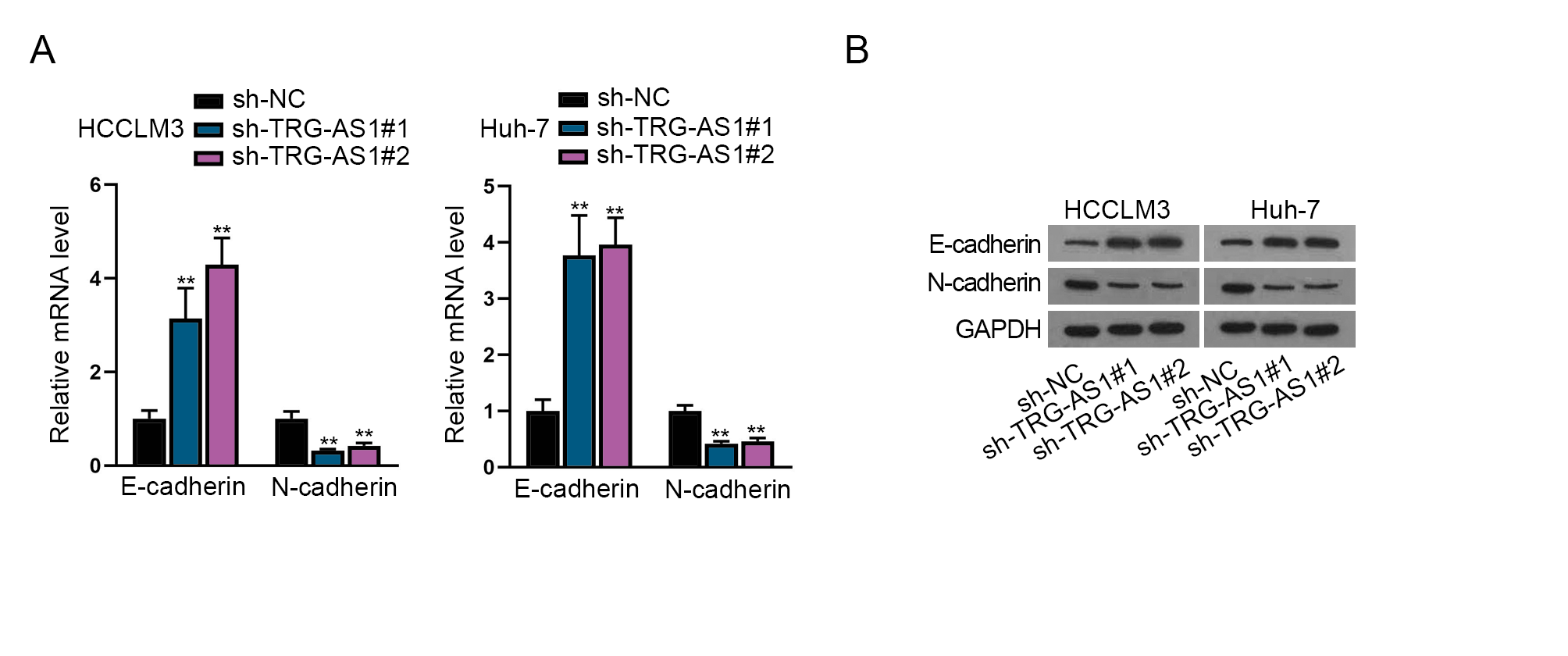

Supplement: Supplementary file 1 — Additional file 1: Figure S1. A. RT-qPCR analysis of E-cadherin and N-cadherin expression in two HCC cells transfected with sh-NC, sh-TRG-AS1#1, sh-TRG-AS1#2. B. Western blot analysis was utilized to examine the protein levels of E-cadherin and N-cadherin in HCC cells transfected with sh-NC, sh-TRG-AS1#1, sh-TRG-AS1#2. **P < 0.01. [file 12935_2020_1440_MOESM1_ESM.tif]

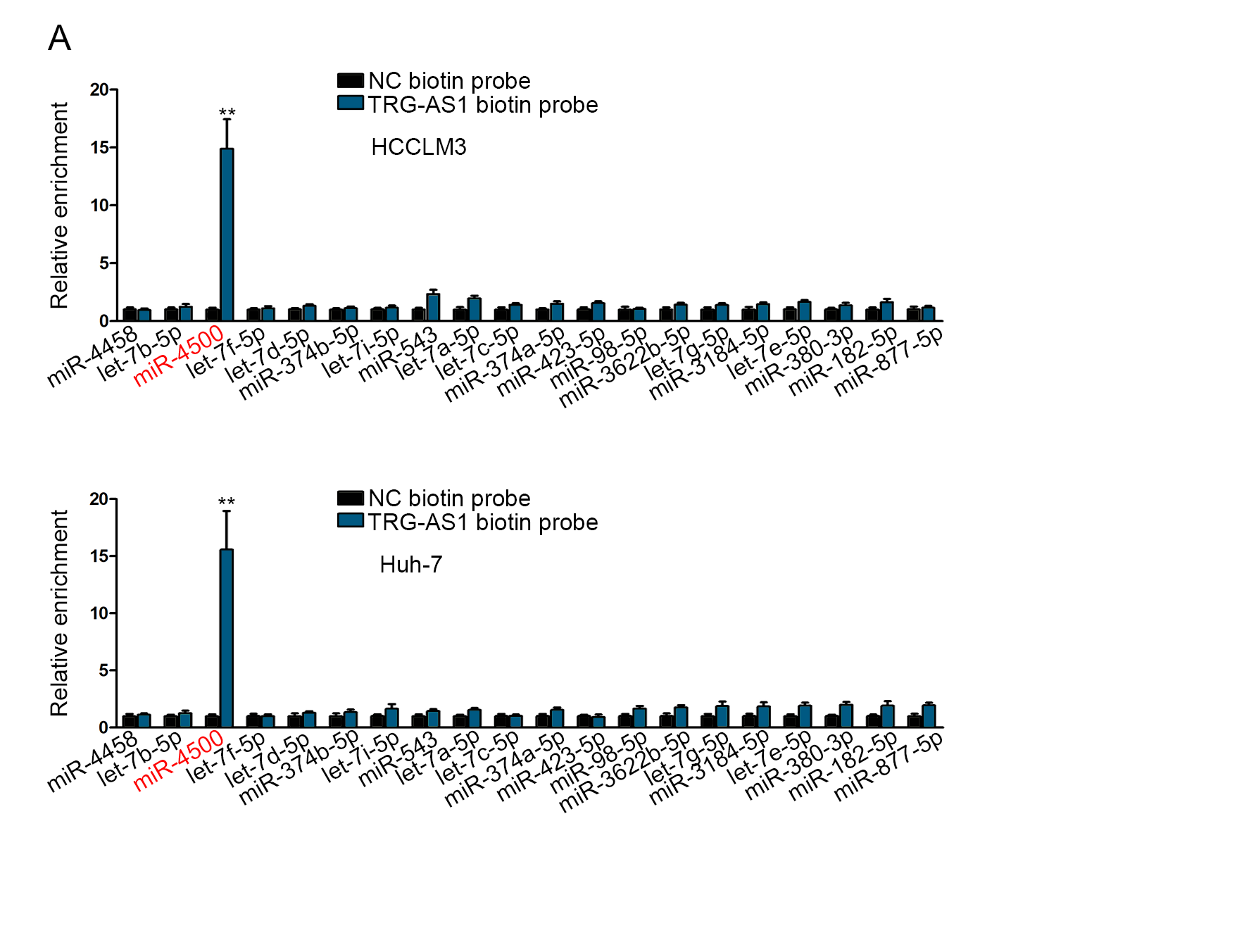

Supplement: Supplementary file 2 — Additional file 2: Figure S2. A. RNA pull down assay was performed using TRG-AS1 biotin probe and enrichment of mRNAs in both HCC cells was analyzed by RT-qPCR. **P < 0.01. [file 12935_2020_1440_MOESM2_ESM.tif]

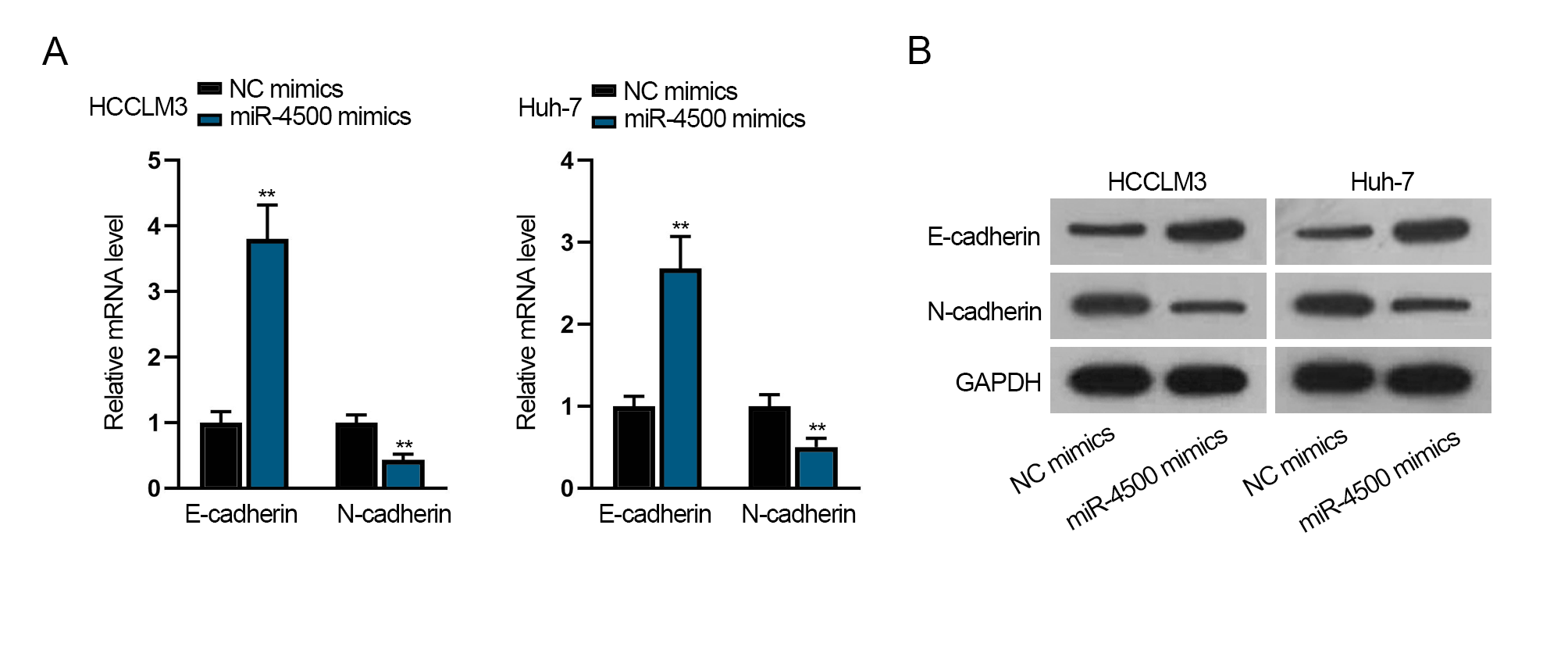

Supplement: Supplementary file 3 — Additional file 3: Figure S3. A-B. The mRNA and protein levels of E-cadherin and N-cadherin were detected by RT-qPCR and western blot analyses in cells with ectopic expression of miR-4500. **P < 0.01. [file 12935_2020_1440_MOESM3_ESM.tif]

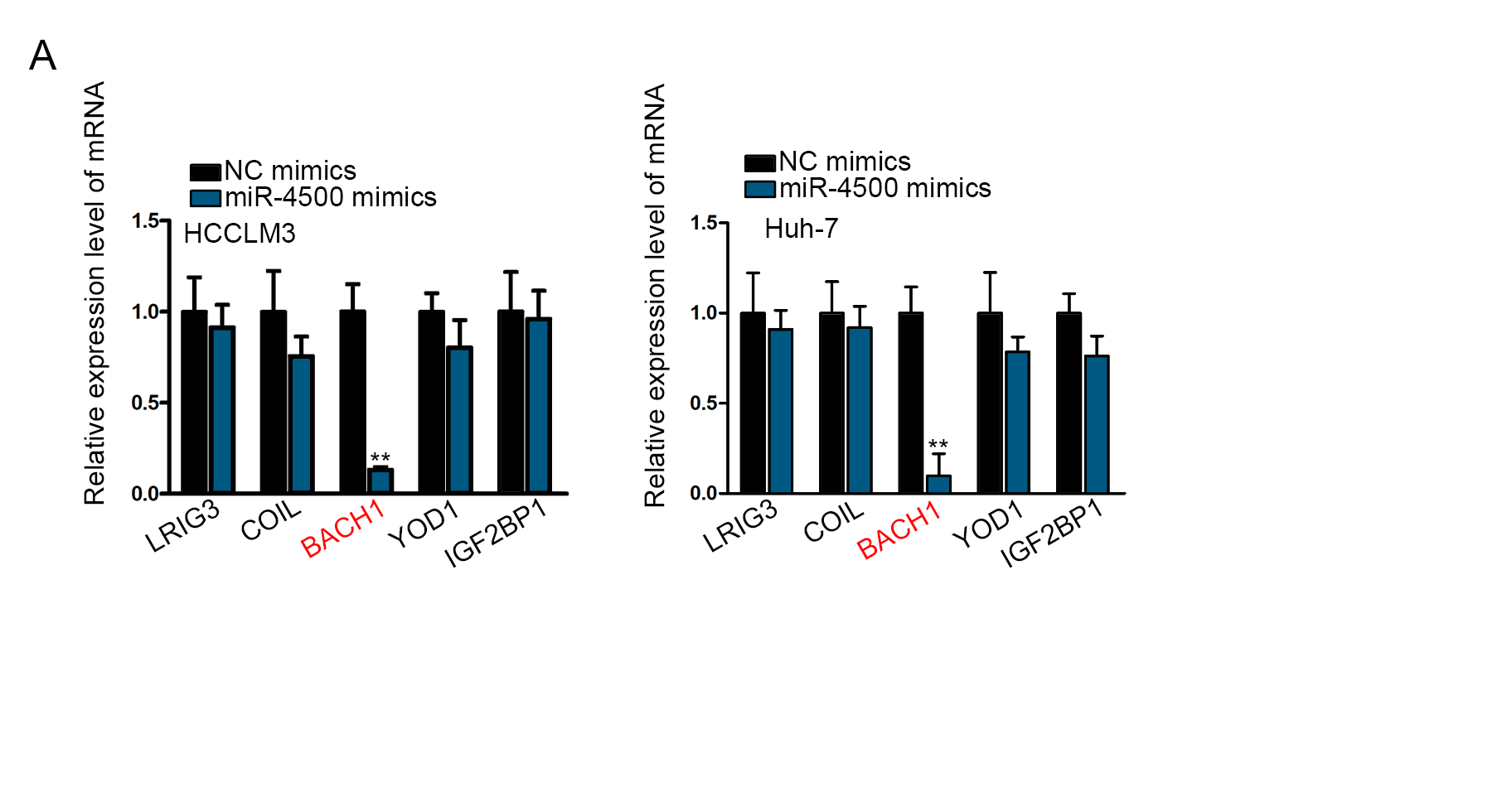

Supplement: Supplementary file 4 — Additional file 4: Figure S4. A. The levels of candidate mRNAs were examined by RT-qPCR in HCCLM3 and Huh-7 cells transfected with miR-4500 mimics. **P < 0.01. [file 12935_2020_1440_MOESM4_ESM.tif]

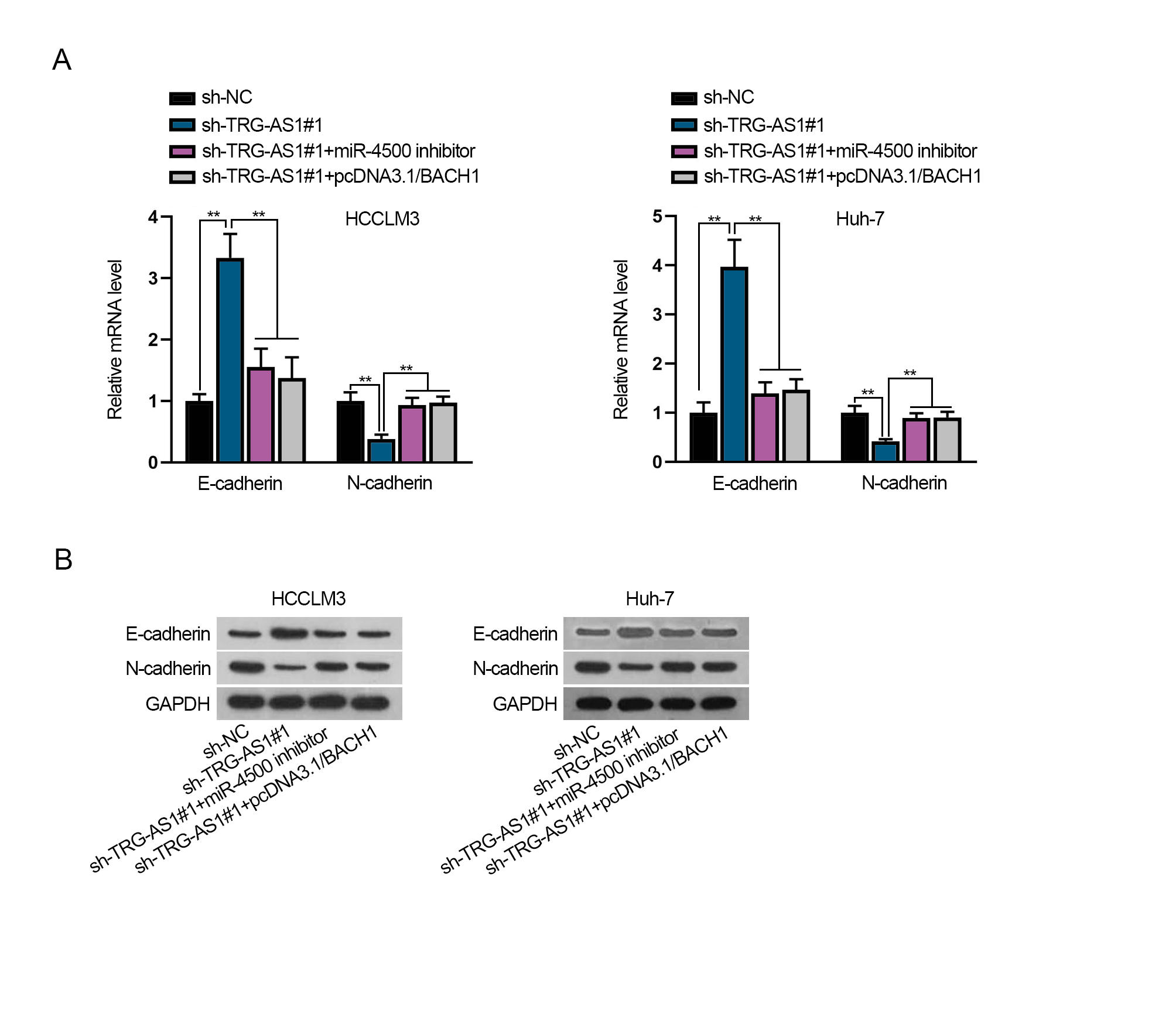

Supplement: Supplementary file 5 — Additional file 5: Figure S5. A-B. The mRNA and protein levels of EMT-makers (E-cadherin and N-cadherin) were measured in indicated HCC cells by RT-qPCR and western blot analyses, respectively. [file 12935_2020_1440_MOESM5_ESM.tif]
